# Data Partitions, Bayesian Analysis and Phylogeny of the Zygomycetous Fungal Family *Mortierellaceae*, Inferred from Nuclear Ribosomal DNA Sequences

**DOI:** 10.1371/journal.pone.0027507

**Published:** 2011-11-10

**Authors:** Tamás Petkovits, László G. Nagy, Kerstin Hoffmann, Lysett Wagner, Ildikó Nyilasi, Thasso Griebel, Domenica Schnabelrauch, Heiko Vogel, Kerstin Voigt, Csaba Vágvölgyi, Tamás Papp

**Affiliations:** 1 Department of Microbiology, Faculty of Science and Informatics, University of Szeged, Szeged, Hungary; 2 Jena Microbial Resource Collection, Department of Microbiology and Molecular Biology, School of Biology and Pharmacy, Institute of Microbiology, University of Jena, Jena, Germany; 3 Department of Molecular and Applied Microbiology, Leibniz–Institute for Natural Product Research and Infection Biology (HKI), Jena, Germany; 4 Department of Bioinformatics, School of Mathematics and Informatics, Institute of Informatics, University of Jena, Jena, Germany; 5 Department of Entomology, Max Planck Institute for Chemical Ecology, Jena, Germany; University of California Riverside, United States of America

## Abstract

Although the fungal order Mortierellales constitutes one of the largest classical groups of Zygomycota, its phylogeny is poorly understood and no modern taxonomic revision is currently available. In the present study, 90 type and reference strains were used to infer a comprehensive phylogeny of Mortierellales from the sequence data of the complete ITS region and the LSU and SSU genes with a special attention to the monophyly of the genus *Mortierella*. Out of 15 alternative partitioning strategies compared on the basis of Bayes factors, the one with the highest number of partitions was found optimal (with mixture models yielding the best likelihood and tree length values), implying a higher complexity of evolutionary patterns in the ribosomal genes than generally recognized. Modeling the ITS1, 5.8S, and ITS2, loci separately improved model fit significantly as compared to treating all as one and the same partition. Further, within-partition mixture models suggests that not only the SSU, LSU and ITS regions evolve under qualitatively and/or quantitatively different constraints, but that significant heterogeneity can be found within these loci also. The phylogenetic analysis indicated that the genus *Mortierella* is paraphyletic with respect to the genera *Dissophora*, *Gamsiella* and *Lobosporangium* and the resulting phylogeny contradict previous, morphology-based sectional classification of *Mortierella*. Based on tree structure and phenotypic traits, we recognize 12 major clades, for which we attempt to summarize phenotypic similarities. *M. longicollis* is closely related to the outgroup taxon *Rhizopus oryzae*, suggesting that it belongs to the Mucorales. Our results demonstrate that traits used in previous classifications of the Mortierellales are highly homoplastic and that the Mortierellales is in a need of a reclassification, where new, phylogenetically informative phenotypic traits should be identified, with molecular phylogenies playing a decisive role.

## Introduction

The order Mortierellales constitutes one of the largest groups of Zygomycota. Most of the taxa are oligo– or mesotrophic and occur typically as saprobes in soil, dung or other decaying organic material. Many of them, such as *Mortierella alpina* and related species, are able to convert various carbon sources into lipids and are of great biotechnological potential as producers of polyunsaturated fatty acids [1]–[3], while others are used as biotransforming agents of various organic compounds [4], [5]. The genus *Mortierella* also contains an animal pathogen, *M*. *wolfii*, which differs from the other species in its thermophilic nature [6].

**Figure 1:**
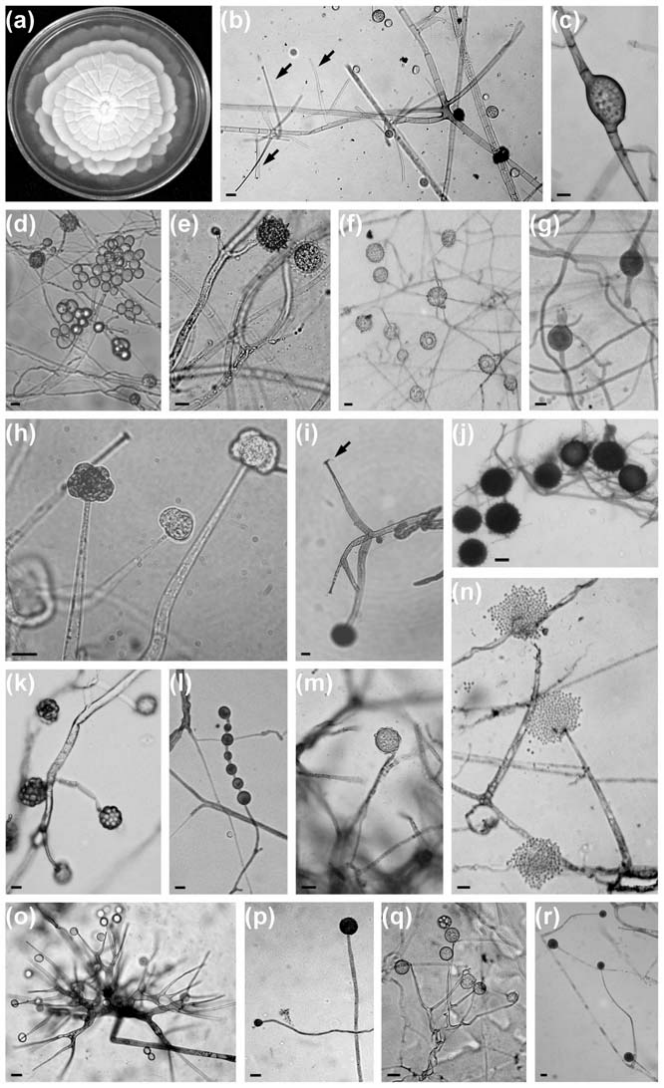
Examples for the macro- and micromorphology of the investigated Mortierellales strains. Lobed growth of *Mortierella capitata* on MEA. (a). Micromorphology of *M. verticillata* (b), arrows indicate the sporangiophores after dehiscence. Chlamydospore of *M. gemmifera* (c). Sporangiophores with sporangiospores (d) and stylospores (e) of *M. polycephala*. Stylospores of *M. polygonia* produced on MEA (f) and its chlamydospores produced on OA (g). Sporangiophores and sporangia of *M. amoeboidea* (h). Sporangiophores of *M. biramosa* (i), the arrow indicates the characteristic collar remaining at the apex after the dehiscence of the sporangium. Stylospores of *M. indohii* (j). Sporangia of *Dissophora decumbens* (k). Chlamydospores (l) and sporangium (m) produced by *D. ornata*. Sporangiophores and sporangia after the dehiscence of *M. turficola* (n). Branching sporangiophores and sporangiospores of *Gamsiella multidivaricata* (o). Sporangia and sporangiophores produced by *M. mutabilis* on MEA (p) and its branching sporangiophores produced on OA (q). Sporangia of *M. exigua* (r). In each panel, the lengths of scale bars denote 10 µm.

Members of the order generally form unusually delicate, cottony mycelia, which are coenocytic when young, but often become septate on ageing. In most cases, the colonies are white to grayish–white and display a rosette–like (zonate) surface on several types of medium (Fig. 1a), pure colonies may produce a characteristic garlic–like odor. These fungi produce sporangia or sporangiola, where columellae are often absent or rudimentary and never protrude into the sporangium, as well as collarettes (Fig. 1i), collar–like structures on the sporangiophores, which are formed by remnants of the sporangial envelope [7]–[9]. The lack of pronounced columellae and the production of non–apophysate sporangia distinguish members of the Mortierellales from those of the Mucorales [10]. They may form smooth or ornamented, intercalary and/or terminal chlamydospores (Fig. 1c, e–g, j, l). Zygospores (sexually produced spores), when present, are formed between apposed suspensors and are often covered by a thick hyphal sheath [6], [9]. As zygospores have been observed for only some of the species [11], the characterization of Mortierellales is based entirely on asexual characteristics [12].

The phylogeny of Mortierellales is poorly understood and no modern taxonomic revision of the order is available. Originally, this fungal group was considered as a family of the Mucorales, named Mortierellaceae [13] containing two genera, *Herpocladium* [14] and *Mortierella* [15]. Subsequently, several genera were added to the family or segregated from the genus *Mortierella*, such as *Dissophora*, *Haplosporangium* [16], *Azygozygum* [17], *Umbelopsis* [18], *Aquamortierella* [19], *Echinosporangium* [20], *Actinomortierella* [21], *Modicella* and *Gamsiella* [22]. *Echinosporangium* is now regarded as a synonym of *Lobosporangium* [22]. The order Mortierellales was proposed by Cavalier–Smith [23] and molecular phylogenetic studies based on ribosomal and protein coding gene sequences reinforced the need for the separation of the Mortierellaceae from the Mucorales [8], [24]–[28].

Although several studies have been performed in attempt to clarify the relationships of zygomycete fungi at the family and ordinal levels, the lack of information concerning the phylogeny of the largest genus, *Mortierella*, is surprising. At present, Mortierellales contains one family, Mortierellaceae, which comprises six genera, *Aquamortierella*, *Dissophora* (Fig. 1k–m), *Gamsiella* (Fig. 1o), *Lobosporangium*, *Modicella* and *Mortierella* [8], [9]. Among them, *Dissophora* and *Modicella* contain only two species each, while *Aquamortierella*, *Lobosporangium* and *Gamsiella* are monotypic. At the same time, *Mortierella* is considered the largest genus of Zygomycota, with about 100 recognized species [9]. Earlier, two subgenera, *Mortierella* and *Micromucor*, were distinguished within it [29]. Based on ITS sequence data, members of the *Micromucor* subgenus, which was also known as the *Mortierella isabellina* group, were transferred to the genus *Umbelopsis* and placed in a newly proposed family, Umbelopsidaceae, within the order Mucorales [30]. Several molecular phylogenetic studies have shown that *Umbelopsis* form a basal sister–group to the Mucorales [8], [24]–[26]. Based on morphological similarities and previous classifications, Gams [29] divided the subgenus *Mortierella* into the following nine sections: *Simplex, Alpina, Schmuckeri, Mortierella, Actinomortierella, Hygrophila, Stylospora, Spinosa* and *Haplosporangium*. In view of the differences and contradictions that can be found among the different classifications [7], [29], [31], it appears reasonable to revise the taxonomy of the genus, which requires an exploration of its phylogenetic structure.

The aim of the present study was to infer the phylogeny of the order Mortierellales from sequence data of three nuclear ribosomal regions (the complete ITS region and the LSU and the SSU genes) with special emphasis on the monophyly of *Mortierella* with respect to *Dissophora*, *Gamsiella* and *Lobosporangium*, since this has been brought into question by preliminary analyses [8]. Hence, ribosomal genes from 90 strains including representatives of the genera *Dissophora*, *Gamsiella*, *Lobosporangium*, *Mortierella* and *Umbelopsis* were sequenced, and phylogenetic analyses involving partitioned and mixture models were undertaken. The monophyly of previously morphologically distinguished sections of *Mortierella* were also tested on the basis of the resulting phylogeny.

Finding the balance between model complexity and the variance of the estimation, or the ease of convergence in Bayesian MCMC analyses is an important and highly debated aspect in phylogenetics [32]–[36]. While it is evident that oversimplified models lead to severely biased estimates, including unreliable posterior probabilities, the effects of overly complex models is not as straightforward. Advocating the power of Bayesian estimation for converging to the right posterior distribution under complex parameter spaces, several recent studies have used highly partitioned complex models (e.g. [35], [37], [38]) and simulation studies support the legibility of highly complex models in molecular evolution. On the other hand, overpartitioned models have been shown to negatively affect MCMC convergence [32], [39] and the computation of Bayes Factors on the basis of harmonic mean likelihoods results in a strong preference of overpartitioned models. How partition boundaries should optimally be defined, and how optimal partitioning relates to commonly recognized, biologically meaningful features within the phylogenetic markers represents an intriguing question with very few experimental analyses so far [35]. Therefore, in this study we examined the effects of increasing model complexity in nearly contiguous spans of the nuclear ribosomal SSU, ITS1, 5.8S, ITS2 and LSU genes and the relationship between *a priori* defined biologically recognized partition boundaries and another partitioning regime, in which homogeneously evolving sites are identified during the MCMC runs [40].

## Results

### Model testing of single gene alignments and tests of congruence

Substitution models for the single–gene alignments were selected by using jModelTest based on the sample–size corrected AIC criterion. When the model suggested by the program was not available in subsequently used software (MrBayes, BayesPhylogenies and RaxML), the next more complex model was chosen for the alignment. The GTR+ Γ substitution model was therefore selected for all single–gene alignments for subsequent analyses. Since we did not find any significant conflict between the single–gene alignments, all the data were concatenated and subjected to supermatrix analyses. The concatenated alignment is available on TreeBase (accession no. : S11367).

### Evaluation of alternative partitioned models

**Figure 2:**
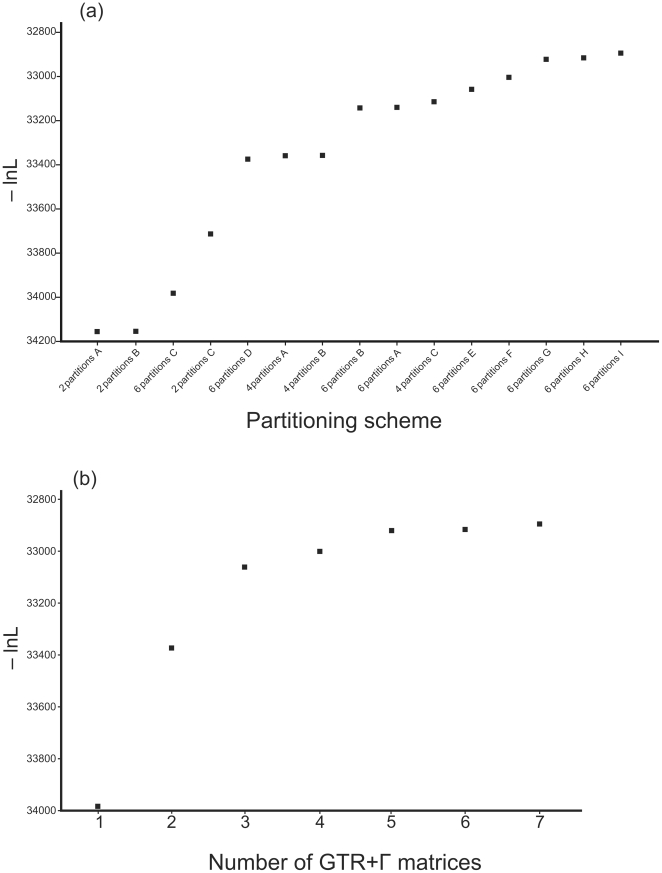
Saturation of log likelihood (lnL) values as a function of model complexity, calculated as a mean of the likelihoods of post–burn–in trees. (a) Comparison of all 15 partitioned models. Figure shows that the indel matrix is best described by one–parameter models, which show up as local plateaus (see arrowheads) in the saturation of likelihoods. (b) Comparison of MCMC analyses performed by using the mixture models (in BayesPhylogenies) only with 1 to 7 GTR+ Γ matrices.

Altogether 15 different partitioned models were compared on the basis of Bayes factors. The Bayesian runs converged quickly to the stationary distributions, well before the specified burn–in values. Paired MrBayes and BayesPhylogenies runs converged to the same region of the posterior based on Bayes factor tests. The mean of the obtained log likelihood values and Bayes factors of pairwise model comparisons are presented in Table 1. As expected, the more complex models fitted the data better in all cases, but the Bayes factor tests revealed an interesting pattern. Increasing the number of partitions always caused significant improvements in the likelihood values (Fig. 2a). Analyses in which the three ribosomal regions (ITS, SSU and LSU) were treated as one contiguous partition and the indel matrix as a second one (“2 partitions A”, “B” and “C”) returned the poorest likelihoods of all runs, regardless of the model used for the specific partitions (conventional GTR+ Γ in “A” and “B” and mixture model in “C”) and are significantly rejected against more sophisticated partitioning regimes. Runs with ITS, SSU and LSU as three partitions returned transitional likelihood scores (“3 partitions A” to “C”), whereas the best results were obtained when the ITS1, 5.8S and ITS2 genes were modeled separately in addition to the SSU, LSU and indel matrices (“6 partitions A” to “I”). It is interesting that increasing the number of parameters for the indel matrix had a negligible effect on the likelihoods (log BF: 0.315–1.369, partitioning schemes “A” versus “B”).

**Table 1:** Comparison of alternative partitioning regimes on the basis of Bayes factors.

Partitioned Model	–lnL	95% HPD	2 partitions A	2 partitions B	6 partitions C	2 partitions C	6 partitions D	4 partitions A	4 partitions B	6 partitions B	6 partitions A	4 partitions C	6 partitions E	6 partitions F	6 partitions G	6 partitions H	6 partitions I	TL
2 partitions A	34155.6	+/− 0.28	–															7.51
2 partitions B	34154.7	+/− 0.26	0.4	–														7.48
6 partitions C	33982.8	+/− 1.00	75.0	74.7	–													10.76
2 partitions C	33711.3	+/− 1.73	192.9	192.5	117.9	–												7.13
6 partitions D	33373.2	+/− 1.59	339.8	339.4	264.7	146.9	–											15.78
4 partitions A	33358.8	+/− 0.39	346.0	345.7	271.0	153.1	6.3	–										8.47
4 partitions B	33358.0	+/− 0.42	346.4	346.0	271.3	153.4	6.6	0.3	–									8.33
6 partitions B	33142.2	+/− 0.27	440.1	439.7	365.0	247.2	100.3	94.0	93.7	–								10.32
6 partitions A	33139.1	+/− 0.29	441.4	441.1	366.4	248.5	101.7	95.4	95.1	1.4	–							10.18
4 partitions C	33114.3	+/− 1.84	452.2	451.8	377.2	259.3	112.4	106.2	105.9	12.2	10.8	–						16.20
6 partitions E	33058.4	+/− 1.04	476.5	476.1	401.5	283.6	136.7	130.5	130.1	36.4	35.1	24.3	–					19.54
6 partitions F	33003.7	+/− 0.98	500.2	499.9	425.2	307.3	160.5	154.2	153.9	60.2	58.8	48.0	23.7	–				20.63
6 partitions G	32923.1	+/− 2.09	535.2	534.9	460.2	342.3	195.5	189.2	188.9	95.2	93.8	83.0	58.7	35.0	–			21.3
6 partitions H	32915.6	+/− 2.04	538.5	538.1	463.4	345.6	198.7	192.4	192.1	98.4	97.0	86.3	62.0	38.2	3.2	–		21.14
6 partitions I	32894.6	+/− 2.48	547.6	547.2	472.6	354.7	207.8	201.6	201.3	107.5	106.2	95.4	71.1	47.4	12.4	9.1	–	22.36

The means of the ln likelihood values (−lnL), the 95% highest posterior densities (HPD) and the tree lengths (TL) averaged over all post–burn–in samples are also shown.

Of the analyses with 6 partitions, better results were obtained when mixture models were used to model the specific partitions. Increasing the number of GTR+ Γ matrices caused improvements in the likelihoods, but the improvements between subsequent models decreased until the likelihoods became saturated (Fig. 2b). This saturation was observable when 5 GTR+ Γ matrices were fitted to the data. Six and seven such matrices did not improve the results appreciably, whereas the computational burden for adding extra matrices to the model increased linearly. Therefore, in subsequent Bayesian analyses we used the “6 partitions G” partitioning and modeling regime as optimal for our data.

It is noteworthy that the total tree lengths recovered by the models also increased when the complexity of partitioning and modeling was increased (Table 2), which suggests that more complex models are able to recover more hidden substitutions than simpler ones. Exceptions from this were “6 partitions C” and “D” which returned much higher TL values than would be expected on the basis of their likelihood–based ranking. This might be caused by differences in branch–length priors between MrBayes and BayesPhylogenies and/or being trapped in regions of the posterior with unrealistically long branches due to poor convergence [41]–[43].

**Table 2:** Compositions of partitioned models tested by Bayesian MCMC analyses of the concatenated alignment.

Partitioning	ITS1	5.8S	ITS2	LSU	SSU	Indel matrix	Software^a^	No. of GTR+ Γ matrices
2 partitions A			GTR+ Γ			Mk1	MB	1
4 partitions A		GTR+ Γ		GTR+ Γ	GTR+ Γ	Mk1	MB	1
6 partitions A	GTR+ Γ	GTR+ Γ	GTR+ Γ	GTR+ Γ	GTR+ Γ	Mk1	MB	1
2 partitions B			GTR+ Γ			Mk2	MB	1
4 partitions B		GTR+ Γ		GTR+ Γ	GTR+ Γ	Mk2	MB	1
6 partitions B	GTR+ Γ	GTR+ Γ	GTR+ Γ	GTR+ Γ	GTR+ Γ	Mk2	MB	1
2 partitions C			GTR+ Γ			Nst1	BP	1
4 partitions C		GTR+ Γ		GTR+ Γ	GTR+ Γ	Nst1	BP	1
6 partitions C	GTR+ Γ	GTR+ Γ	GTR+ Γ	GTR+ Γ	GTR+ Γ	Nst1	BP	1
6 partitions D	GTR+ Γ	GTR+ Γ	GTR+ Γ	GTR+ Γ	GTR+ Γ	Nst1	BP	2
6 partitions E	GTR+ Γ	GTR+ Γ	GTR+ Γ	GTR+ Γ	GTR+ Γ	Nst1	BP	3
6 partitions F	GTR+ Γ	GTR+ Γ	GTR+ Γ	GTR+ Γ	GTR+ Γ	Nst1	BP	4
6 partitions G	GTR+ Γ	GTR+ Γ	GTR+ Γ	GTR+ Γ	GTR+ Γ	Nst1	BP	5
6 partitions H	GTR+ Γ	GTR+ Γ	GTR+ Γ	GTR+ Γ	GTR+ Γ	Nst1	BP	6
6 partitions I	GTR+ Γ	GTR+ Γ	GTR+ Γ	GTR+ Γ	GTR+ Γ	Nst1	BP	7

a MB: MrBayes 3.1.2, BP: BayesPhylogenies 1.0.

### Phylogenetic analyses and relationships within the Mortierellales

**Figure 3:**
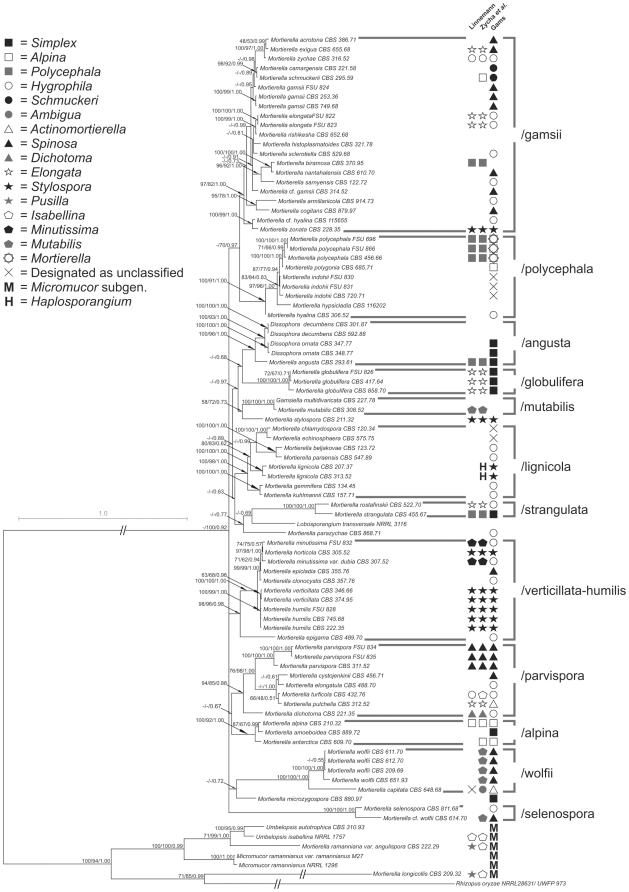
The 50% Majority Rule consensus phylogram inferred from the nuclear ribosomal sequence data of 90 Mortierellales strains using BayesPhylogenies, under a model with 5 GTR+ Γ matrices for each of the six partitions. Numbers above branches indicate Maximum Parsimony (MPBS), Maximum Likelihood bootstrap (MLBS) percentages and Bayesian posterior probabilities (BBP), respectively. Missing or weakly supported nodes (<50% or 0.5) are denoted by a “–”.

All three replicates of the final BayesPhylogenies analysis converged to the same posterior distribution, hence, the trees were pooled and a consensus tree was computed. The majority rule consensus tree computed from 2×10^5^ trees sampled after stationarity is presented in Fig. 3 with MPBS, MLBS and BPP values on the branches.

At the genus level, two large clades can be recognized, *Umbelopsis* (MPBS: 100%, MLBS: 100%, BPP: 0.99) and *Mortierella* (MPBS: –, MLBS: 100%, BPP: 0.92). As expected, *Umbelopsis,* including representatives of *Micromucor,* clusters with *Rhizopus oryzae*, a representative of the Mucorales. The genera *Dissophora*, *Lobosporangium* and *Gamsiella* are nested within the genus *Mortierella* with significant support. With the exception of *Mortierella longicollis*, a monophyletic Mortierellaceae can be recognized if the above–mentioned genera are included. *M. longicollis* is placed in a basal position, closest to the outgroup taxon *R. oryzae* (Mucorales).

Within the *Mortierella* clade, 12 large clades were distinguished and named after a representative or well–known species of the genus. The /selenospora clade (MPBS: 100%, MLBS: 100%, BPP: 1.00) includes the *M. wolfii* strain CBS 614.70 and the type strain of *M. selenospora*. The position of the other isolates of *M. wolfii* suggests that strain CBS 614.70 was misidentified, which is reinforced by the mesophilic nature of this strain with an optimal growth temperature of 24 °C. The thermophilic *M. wolfii* isolates were found in the /wolfii clade (MPBS: 100%, MLBS: 100%, BPP: 1.00) together with *M. capitata*. The /alpina clade (MPBS: 100%, MLBS: 92%, BPP: 1.00) contains three closely related taxa, *M. alpina*, *M. amoeboidea* and *M. antarctica,* all with preference for low temperatures. The /parvispora clade (MPBS: 94%, MLBS: 85%, BPP: 0.88) contains six taxa, of which *M. cystojenkinii*, *M. elongatula*, *M. turficola* and *M. pulchella* form a closely related subclade. The /verticillata–humilis clade (MPBS: 98%, MLBS: 96%, BPP: 0.98) includes eight taxa, among which isolates of *M. verticillata* and *M. humilis* form one strongly supported (MPBS: 100%, MLBS: 99%, BPP: 1.00) species–level group. *M. minutissima* and *M. horticola* also seem to be very closely related. The /strangulata clade (MPBS: 100%, MLBS: 100%, BPP: 1.00) contains *M. rostafinski* and *M. strangulata*. *Lobosporangium transversale* is found as a sister group of this clade with low support (MPBS: –, MLBS: –, BPP: 0.69). The seven species in the well–defined /lignicola clade (MPBS: 100%, MLBS: 98%, BPP: 1.00) are distributed in four similarly well–supported subclades. The /globulifera clade (MPBS: 100%, MLBS: 100%, BPP: 1.00) only includes representatives of *M. globulifera*. The /angusta (MPBS: 100%, MLBS: 96%, BPP: 1.00) and /mutabilis (MPBS: 100%, MLBS:100%, BPP: 1.00) clades contain the two non-mortierellalean genera, where *Dissophora* belongs to the former and *Gamsiella* to the latter. The type species of the genus *Mortierella*, *M. polycephala*, is included in the /polycephala clade (MPBS: 100%, MLBS: 91%, BPP: 1.00), together with *M. polygonia*, *M. indohii*, *M. hypsicladia* and *M. hyalina*. The most species–rich and heterogeneous group is the /gamsii clade (MPBS: 97%, MLBS: 82%, BPP: 1.00), consisting of several species and subgroups.

The gross topology and strongly supported nodes of the trees were affected neither by GBlocks-curation of the ITS alignment nor the exclusion of the complete ITS1-5.8S-ITS2 region or the indel-matrix (Fig. S1, S2, S3, S4). Table 3 shows the posterior probability values of the 12 main clades inferred from the different analyses. Because the different exclusion strategies have not influenced the resolvability of the backbone of the tree, it is likely that the polytomies there were not caused by intra-alignment conflict or alignment noise. However, the position of some clades varied between the analyses. The position of two out of the 12 larger clades identified in this study (Fig. 3) seems to be dependent on the inclusion and (partial) exclusion of the ITS locus and the indel-matrix, although neither of these were strongly supported in the original analyses. When the ambiguously aligned sites were excluded, the /strangulata clade was nested between the /angusta and /mutabilis clades with low support (Fig. S1, S2), although the position of the /strangulata clade was also weakly supported on the basis of the original dataset (Fig. 3). Similarly, the /selenospora clade is collapsed to polytomy on the consensus tree inferred from the original dataset, while it is resolved as the sister clade of the /polycephala clade when the indel-matrix was excluded. However, statistical support for the latter position is lacking (0.80 and 0.83, Fig. S2, S3).

**Table 3:** Bayesian posterior probability values of the 12 larger *Mortierella* clades inferred from different datasets.

	ITS-nrLSU-nrSSU-gap	gbITS-nrLSU-nrSSU-gap	gbITS-nrLSU-nrSSU	nrLSU-nrSSU-gap	nrLSU-nrSSU
/gamsii	1.00	0.96	1.00	1.00	1.00
/polycephala	1.00	1.00	1.00	1.00	1.00
/angusta	1.00	1.00	0.70	1.00	1.00
/globulifera	1.00	1.00	1.00	1.00	1.00
/mutabilis	1.00	1.00	1.00	1.00	1.00
/lignicola	1.00	1.00	1.00	1.00	1.00
/strangulata	1.00	1.00	1.00	1.00	1.00
/verticillata-humilis	1.00	1.00	1.00	1.00	1.00
/parvispora	1.00	1.00	1.00	1.00	0.61
/alpina	1.00	1.00	0.94	1.00	0.95
/wolfii	1.00	1.00	1.00	1.00	1.00
/selenospora	1.00	1.00	1.00	1.00	1.00

## Discussion

### Model selection

For the data set used here, we observed increases in the likelihood values when the complexity of the model was increased. Although this accords with expectations based on the properties of likelihood approaches, it has important implications for the handling of ribosomal genes in phylogenetic analyses. There has been some concern regarding the independence of ribosomal loci and the evolutionary processes acting on them (apart from concerted evolution). Some authors argue that the SSU, ITS and LSU genes are not independent and, among others, cannot be listed as different loci in phylogeny papers [44]. We found that separate modeling of the three regions improved the fit of the model to the data significantly, which suggests that the three loci evolve not only at different rates, but also under different constraints. In addition, we found that dividing the ITS regions into three partitions, ITS1, 5.8S and ITS2, also improved the results significantly. We therefore suggest independent modeling of these three regions in phylogenetic analyses when the amount and quality of the phylogenetic signal contained within them can support such parameter–rich models.

We observed extensive rate heterogeneity within the individual loci by using mixture models. These models are able to recover hidden evolutionary patterns in the data by iterating several (1 to 7 in our case) GTR+ Γ matrices for each site of the alignment during the MCMC run. In this manner, each site in the alignment is assigned the most appropriate GTR+ Γ matrix during the analysis, which can be considered a means of automatic partitioning of the alignments. For most of our partitions, five different matrices proved optimal for a description of the underlying patterns of evolution. For ribosomal coding regions, such models can be used as a surrogate for more parameter–rich secondary structure models, such as the doublet model in MrBayes [45]. In such cases, the mixture model does not require prior knowledge of the secondary structure and *a priori* partitioning of the alignment, which is often difficult, especially in groups with poor taxonomic coverage of experimentally established secondary rRNA structures.

It is also noteworthy that the exclusion of the complete ITS region strongly decreases tree resolution, which suggests that the ITS region contributes valuable phylogenetic signal congruent with that in the LSU and SSU genes, which is expected when the inference of positional homologies and indel placement in the ITS locus is accurate (see Fig. S1, S2, S3, S4).

### Phylogenetic relationships

Our study included all genera of Mortierellales accepted to date, with the exceptions of the monotypic *Aquamortierella* and the bitypic *Modicella*, from which no isolates could be obtained. White et al. [8] also reported that these species had never been cultured. The topology of the 50% Majority Rule consensus phylogram obtained in this study and the larger clades identified in this tree are in many cases in conflict with former, morphology–based subdivisions of the Mortierellales and do not fit the sections of the genus *Mortierella* established by different authors [7], [29], [31] (Fig. 3). Species of *Dissophora*, *Gamsiella* and *Lobosporangium* are nested in well–supported *Mortierella* clades, indicating the paraphyly of the latter genus. This result supports the suspicion of White et al. [8] based on a preliminary two–locus phylogenetic analysis. Moreover, both *Dissophora* and *Gamsiella* species were proven to be closely related to certain *Mortierella* species, i.e. *M. angusta* and *M. mutabilis*, respectively. Benjamin originally described *G. multidivaricata* as *M. multidivaricata* [46], as the only member of the newly established subgenus *Gamsiella.* Later, Benny and Blackwell elevated this subgenus to a generic level [22] because of the presence of characteristic, repeatedly divaricately branching sporangiophores (Fig. 1o), two–spored sporangia and terminal, ornamented chlamydospores. *M. mutabilis* was described as a member of the section *Mutabilis* of *Mortierella* with long, monopodially or synpodially branched sporangiophores and rarely observable chlamydospores [7], [31]. Although the strain CBS 308.52, syntype of *M. mutabilis*, showed this morphology on MEA (Fig. 1p), on certain media, such as OA, we observed sporangiophores, sporangia and, what is more, terminal chlamydospores very similar to those of *G. multidivaricata* (Fig. 1q). It is worth mentioning that, our observations indicated that the presence or absence and the shape of the sporangiophores, the sporangia and the chlamydospores depend to a considerable extent on the culturing medium applied (cf. Fig. 1f–g, p–q). For these traits, we demonstrated large differences between closely related species in many cases and high levels of homoplasy across the tree, which suggests that phenotypic characters have been evolving at fast rates in the Mortierellales. Moreover, only one or a few environmental isolates and relatively old morphological descriptions are available for several species, and reliance on these descriptions without explicit information on the culturing conditions may confound comparisons between different descriptions. These facts certainly contribute to the difficulties inherent in the morphology–based identification of Mortierellales species and to the prevalence of misidentifications in this group. We therefore believe that a standardized technique should be developed for the culturing and description of *Mortierella* species.

*M. longicollis* was initially considered a member of the *M. isabellina* group and the *Micromucor* subgenus [7], [29], [31]. With regard to ITS RFLP and sequence data, this group was later transferred into the genus *Umbelopsis* in the Mucorales [30]. However, that analysis found *M. longicollis* falling outside the genus *Umbelopsis* and more closely related to *Mortierella*. In our multilocus phylogeny, this species is situated far from the Mortierellales and also out of the *Umbelopsis* clade, being a sister group of the core Mucorales represented by *Rhizopus oryzae* (Mucorales, Mucoraceae), which suggests that *M. longicollis* is actually a mucoralean fungus, whose taxonomic position demands further analysis and the inclusion of more taxa from the Mucorales.

The ITS sequence of a *M. turficola* strain (GenBank accession no.: AJ878784) led Kwasna et al. [47] to conclude that this species may also belong in *Umbelopsis*. Linnemann [31] and Gams [29] placed *M. turficola* in the section *Hygrophila* of *Mortierella*, while Zycha et al. [7] classified it in the section *Isabellina*. This section contained other species that were later transferred to *Umbelopsis* by Meyer and Gams [30], but they did not include *M. turficola* in their study. In our analysis, the neotype strain of *M. turficola* (Fig. 1n) was found to be closely related to *M. pulchella*, in the /parvispora clade of our tree, indicating that the strain used by Kwasna et al. [47] must have been misidentified.

*M. humilis* and *M. verticillata* (Fig. 1b) form a subclade within the clade /verticillata–humilis. The clade of *M. verticillata* includes syntype strains of *M. humilis* and *M. marburgensis*, which suggests that these three species are conspecific. Sexual compatibility tests between *M. humilis* and *M. verticillata* support this finding [48]. However, the distinction between the two species has been maintained on the basis of the differences in the ornamentation of sporangiospores observed on scanning electron microscopy. We consider that the evidence provided by the phylogenetic structure and the interfertility of *M. humilis* and *M. verticillata* is sufficiently unequivocal for them to be regarded as a single species.

*M. polycephala* (Fig. 1d, e), *M. polygonia* (Fig. 1f, g) and *M. indohii* (Fig. 1j) were earlier placed in various sections [29], whereas our phylogeny has furnished evidence of their high affinity for each other (/polycephala clade). Further, these species exhibit the special feature of producing characteristic terminal, stalked chlamydospores, also known as stylospores (Fig. 1e, j). Relationship of *M. polycephala* and *M. indohii* has already been proposed on the basis of these structures [48]. Similar terminal chlamydospores have been observed in the case of *M. polygonia* (Fig. 1f).

This study has addressed the monophyly of *Mortierella* and the phylogenetic affinities of several segregate genera, such as *Gamsiella*, *Lobosporangium* or *Dissophora* for the first time. We found that a large monophyletic *Mortierella s. str.* clade can be discerned, which contains the type species of the genus *Mortierella*, *M. polycephala* Coem. 1863. The results necessitate either that the above–mentioned genera be placed within the genus *Mortierella* or that several distinct genera should be described in order to achieve a natural classification of the Mortierellales. Our results suggest that the phenotypic traits of *Mortierella* species depend strongly on the culturing conditions, which makes the search for synapomorphic generic criteria difficult. Thus, taxonomic descriptions should follow a standardized procedure for the reporting of the phenotype. We consider the main source of taxonomic disagreement and confusion between earlier monographic treatments of *Mortierella* to be the lack of standards, which is also the major cause of the frequent misidentifications in strain collections and environmental studies [49]. Our results underline the need for a new classification of Mortierellales, where molecular phylogenetic analysis should play a decisive role, and for a careful taxonomic and phylogenetic revision of the described species and sections within the genera.

## Materials and Methods

### Taxon sampling and culturing conditions

A total of 90 strains were obtained from the Centraalbureau voor Schimmelcultures (CBS–KNAW, Utrecht, the Netherlands) and the Jena Microbial Resource Collection (University of Jena, Germany) and examined in this study (Table S1). Based on previously published classifications and phylogenies, 85 strains of the Mortierellales were selected, representing the genera *Mortierella* (61 taxa), *Gamsiella* (1 species), *Dissophora* (2 species) and *Lobosporangium* (1 species). In addition, 4 representatives of the Umbelopsidaceae (*Umbelopsis* and the synonymous *Micromucor*, 2 taxa each) were analyzed. Two sequences of *Rhizopus oryzae* NRRL 28631 were downloaded from GenBank (LSU, AY213626 and SSU, AF113440) and used as outgroup. An attempt was made to obtain the type strain of the respective species, whenever possible. We included multiple specimens of *M. wolfii* and *M. gamsii*, since preliminary analyses split them into different clades (results not shown).

Strains were grown in liquid malt–extract medium (5% malt extract, 1% glucose) for DNA extraction and on malt–extract agar (MEA, 5% malt extract, 1% glucose, 2% agar), oatmeal agar (OA, Difco, Becton, Dickinson, MD, USA) or cornmeal agar (CMA, 6% cornmeal, 1.5% agar) for morphological examinations. Cultivations were performed at 20–37°C for 7–12 days, depending on the requirements of the fungus.

### DNA sequencing

Genomic DNAs were prepared from 10 mg of mycelia ground to a fine powder in liquid nitrogen and purified by using the MasterPure Yeast DNA purification kit (Epicentre, Madison, WI, USA) according to the instructions of the manufacturer. For all strains, ITS, LSU and SSU regions of the nuclear ribosomal rDNA were amplified by PCR, using the following primers: ITS1 and ITS4 for the complete ITS1 – 5.8S – ITS2 region, LR0R and LR7 for the first 1.5 kb of the nuclear LSU gene, and NS1 and NS4 for an approximately 1.0 kb long portion of the nuclear SSU gene [50], [51]. Reactions were performed in a final volume of 20 µl, according to standard protocols [50]. Amplicons were sequenced on an ABI 3730xl automatic DNA sequencer (Applied Biosystems, Carlsbad, CA, USA) from both directions with the same primers, except for the LSU gene, for which the primer LR5 was used in some cases. Individual readings were assembled to contigs by using the PreGap and Gap4 programs of the Staden Package [52]. All sequences have been deposited in GenBank (Table S1).

### Alignments, model testing and tests of congruence

Three alignments were compiled for this study. After the exclusion of non-overlapping leading and trailing gaps, the lengths of the SSU, ITS1 – 5.8S – ITS2 and LSU alignments were 1.018, 1362 and 1.504 bp, respectively, of which the numbers of parsimony informative characters were 218, 470 and 398, respectively. Of the ITS1 – 5.8S – ITS2 regions, the ITS1 contained 659 nucleotide sites, the 5.8S gene contained 165 sites, and the ITS2 contained 538 sites. Gaps in the ITS1 – 5.8S – ITS2 alignment were recoded as a binary partition, resulting in 553 characters, of which 349 were parsimony informative. The concatenated alignment consisted of 3884 nucleic acid sites, plus 553 characters obtained by indel coding.

The alignments of the LSU and the SSU sequences were computed by ClustalX [53], followed by manual refinement where necessary. Because of the high number of indels, for the alignment of the ITS1 – 5.8S – ITS2 sequences we used the Probalign algorithm [54] with default settings. Leading and trailing gaps were deleted from the alignments. Indels in the ITS alignment were recoded as a binary matrix by means of the simple indel coding algorithm [55] as implemented in FastGap 1.21 [56]. This “indel matrix” was used in all Bayesian analyses.

Best–fit substitution models including rate heterogeneity were selected for each alignment, using jModeltest [57], the results of the sample–size corrected Akaike Information Criterion (AIC_c_) being preferred. Models with a proportion of invariant sites (I) in addition to Γ were excluded from the comparisons, since “I” accounts basically for the same phenomenon as “Γ” and the non–identifiability and interdependence of these two parameters have been reported [32], [58].

Congruence of the phylogenetic signals in the single–gene alignments was tested by comparing Maximum Likelihood (ML) trees, using the approximately unbiased test in CONSEL 0.1 [59], [60]. ML trees and single–site likelihoods were estimated from each single–gene alignment in 10 replicates, using the model selected by AIC_c_ in RaxML 7.0.3 [61].

### Evaluation of alternative partitioned models

To identify the best strategy for partitioning our concatenated alignment, we compared a series of partitioned and/or mixture models. Fifteen different partitioned models (Table 2) were set up as follows. Partitioning was designed on the basis of the gene function and borders. The first set of analyses considered the complete ITS region and the partial LSU and SSU genes as the first partition, and the indel matrix as the second partition (analyses “2 partitions A” and “2 partitions B”). Next, ITS1 – 5.8S – ITS2, LSU and SSU were analyzed separately as three genes. These, together with the indel matrix, resulted in four partitions (analyses “4 partitions A” and “4 partitions B”). In the third set of analyses, the ITS1, 5.8S and ITS2 sequences and the LSU and the SSU regions were modeled separately, which resulted in 6 partition data sets, where the sixth partition was the indel matrix (analyses “6 partitions A” and “6 partitions B”). The notations “A” and “B” refer to two series of the above–mentioned analyses, in which the indel matrix was modeled by a one–state and a two–state Markov model, respectively [62].

The data were also analyzed under mixture models, which offer many theoretical advantages over the traditional ones [40]. When these models are applied in an MCMC framework, more than one GTR+ Γ matrix is fitted to each site of the alignment, which enables hidden evolutionary patterns to be captured more efficiently. We determined the optimal number of GTR+ Γ matrices by looking for the saturation of likelihood values when additional GTR+ Γ matrices were added to the model. Analyses with 6 partitions and 1 to 7 GTR+ Γ matrices (“6 partitions C” to “I”) were therefore run. As a means of comparison between traditional and mixture models, data sets “2 partitions C”, “4 partitions C” and “6 partitions C” were run as above, but with 5 GTR+ Γ matrices estimated and compared on the basis of Bayes factors as described below.

These models were evaluated and compared via their marginal likelihoods estimated via Bayesian MCMC. We approximated the marginal likelihoods by using MrBayes 3.1.2 [63] and BayesPhylogenies 1.0 [64]. Each analysis was run for 4×10^6^ generations, with a conservative burn–in value of 3×10^6^ generations. Model parameters (statefreq, revmat and shape) were unlinked between partitions. For each gene, the substitution model selected by AIC_c_ in jModelTest was invoked. In the case of the indel matrix, a correction for constant characters not included in the matrix was invoked. We sampled one in every 1000 generations. When completed, parameters files were imported in Tracer 1.4 [65] and Bayes factors were calculated by using the modification proposed by Suchard et al. [66] (Table 1). A logBF value >2 was regarded as positive and one >5 was accepted as strong evidence in favor of a better fitting model. Paired runs in MrBayes and BayesPhylogenies were first compared with each other, if the difference was insignificant, only one was retained for comparison with other models.

### Phylogenetic analyses

Bayesian MCMC, ML bootstrap (MLBS) and Maximum Parsimony bootstrap (MPBS) analyses were performed on the concatenated alignment.

Bayesian inference was performed with the model selected by the Bayes factor tests in BayesPhylogenies. Three replicates of 2×10^7^ generations were run, every 1000th state being saved. For each partition, the model selected by jModelTest (GTR+ Γ) was invoked. For the indel matrix, a one–state Markov model was used, with a correction for invariant sites not included in the matrix. The burn–in value was determined by checking likelihood and topological convergence. The convergence of likelihood values was checked by using Tracer 1.4, while topological convergence was inspected by using AWTY [67]. To obtain posterior probabilities, post–burn–in trees of the three runs were pooled and a 50% Majority Rule phylogram was generated by using the CONSENSE program of the PHYLIP package [68]. Clades receiving Bayesian posterior probabilities (BPPs) ≥0.95 were considered significantly supported.

MLBS analysis was run by using the parallel version of RaxML 7.0.3, with five partitions, each modeled by the GTR+ Γ model of evolution. Gaps were treated as missing data. One thousand nonparametric bootstrap replicates were run. Clades receiving 70% or higher bootstrap support were considered to be significantly supported.

Equally weighted MP searches were executed in PAUP v. 4.0b10 [69] according to the following strategy: initial heuristic searches were performed in 1000 replicates to identify tree islands with saving of a maximum of 5 trees per replicate (nchuck = 5, chuckscore = 1, TBR branch–swapping, MAXTREES set to autoincrease). Subsequently, more thorough branch swapping was conducted on the trees resulting from the search outlined above (start = current, nchuck = 0, chuckscore = 0). Gaps were treated as missing data. Nodal support was estimated through 1000 bootstrap replicates with 10 random sequence additions per replicate.

### Analysis of the exclusion of insertion-deletions in the ITS region

Both, the ITS 1 and ITS2 regions contain a large number of insertions-deletions (indels) which makes them difficult to align. To examine the noise coming from potentially misaligned sites the ITS alignment and the reliability of the indel-matrix, we set up four additional datasets as follows: (1) exclusion of the ambiguously aligned positions of the ITS region but leaving the indel-matrix, (2) exclusion of the ambiguously aligned positions of the ITS region and the indel-matrix, (3) exclusion of the complete ITS1-5.8-ITS2 but keeping the LSU and SSU data together with the indel-matrix, and (4) exclusion of the complete ITS1-5.8-ITS2 and the indel matrix keeping only the LSU and SSU data. Poorly aligned positions were eliminated by using the GBlocks server running version 0.91b of the program [70]. During this process we set the following parameters to the program: "Allow smaller final blocks", "Allow gap positions within the final blocks", and "Allow less strict flanking positions". The four resulting datasets were then analyzed by BayesPhylogenies as described above. Consensus trees were computed for each analysis by using the SumTrees script of the DendroPy package [71] in each case. The resulting trees can be found in the Supplementary Material (Fig. S1–S4).

## Supporting Information

Figure S1 **Consensus tree computed from 4000 post-burn-in trees sampled by using the GBlocks curated ITS region, the nuclear ribosomal large (LSU) and small (SSU) subunits and the indel-matrix.**
(PDF)

Figure S2 **Consensus tree computed from 4000 post-burn-in trees sampled by using the GBlocks curated ITS region, the nuclear ribosomal large (LSU) and small (SSU) subunits without the indel-matrix.**
(PDF)

Figure S3 **Consensus tree computed from 4000 post-burn-in trees sampled by using the nuclear ribosomal large (LSU) and small (SSU) subunits without the indel-matrix.**
(PDF)

Figure S4 **Consensus tree computed from 4000 post-burn-in trees sampled by using the nuclear ribosomal large (LSU) and small (SSU) subunits and the indel-matrix.**
(PDF)

Table S1 Fungal strains included in this study, their collection numbers and type status, and the accession numbers of the sequences deposited in GenBank.
(PDF)
