# Supplementary figures and images for: Data Partitions, Bayesian Analysis and Phylogeny of the Zygomycetous Fungal Family Mortierellaceae, Inferred from Nuclear Ribosomal DNA Sequences
Source: PLoS One. 2011 Nov 10;6(11):e27507. doi: 10.1371/journal.pone.0027507 (PMC3213126; doi:10.1371/journal.pone.0027507)

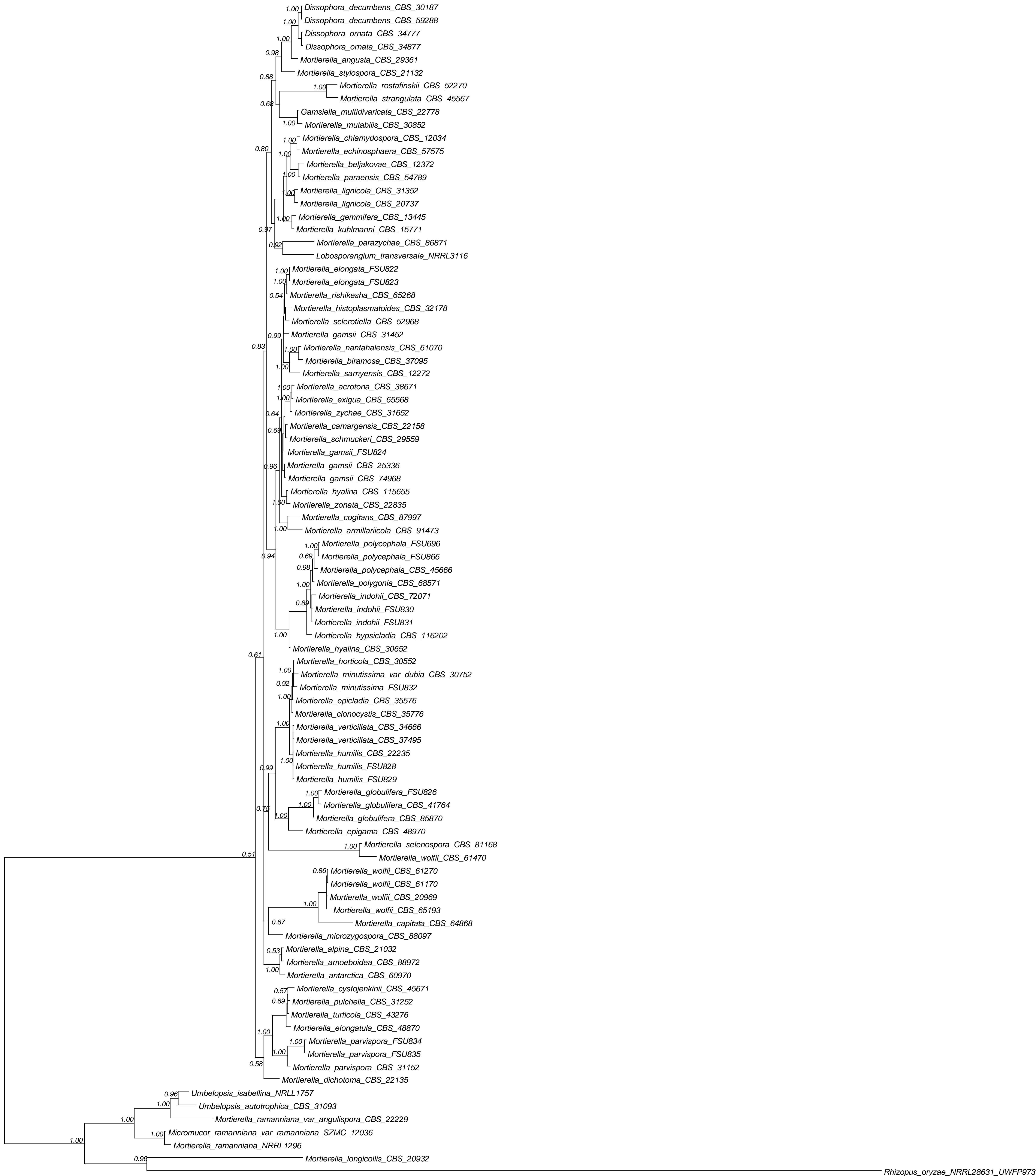

Supplement: Figure S1 — Consensus tree computed from 4000 post-burn-in trees sampled by using the GBlocks curated ITS region, the nuclear ribosomal large (LSU) and small (SSU) subunits and the indel-matrix. (PDF) [file pone.0027507.s001.pdf]

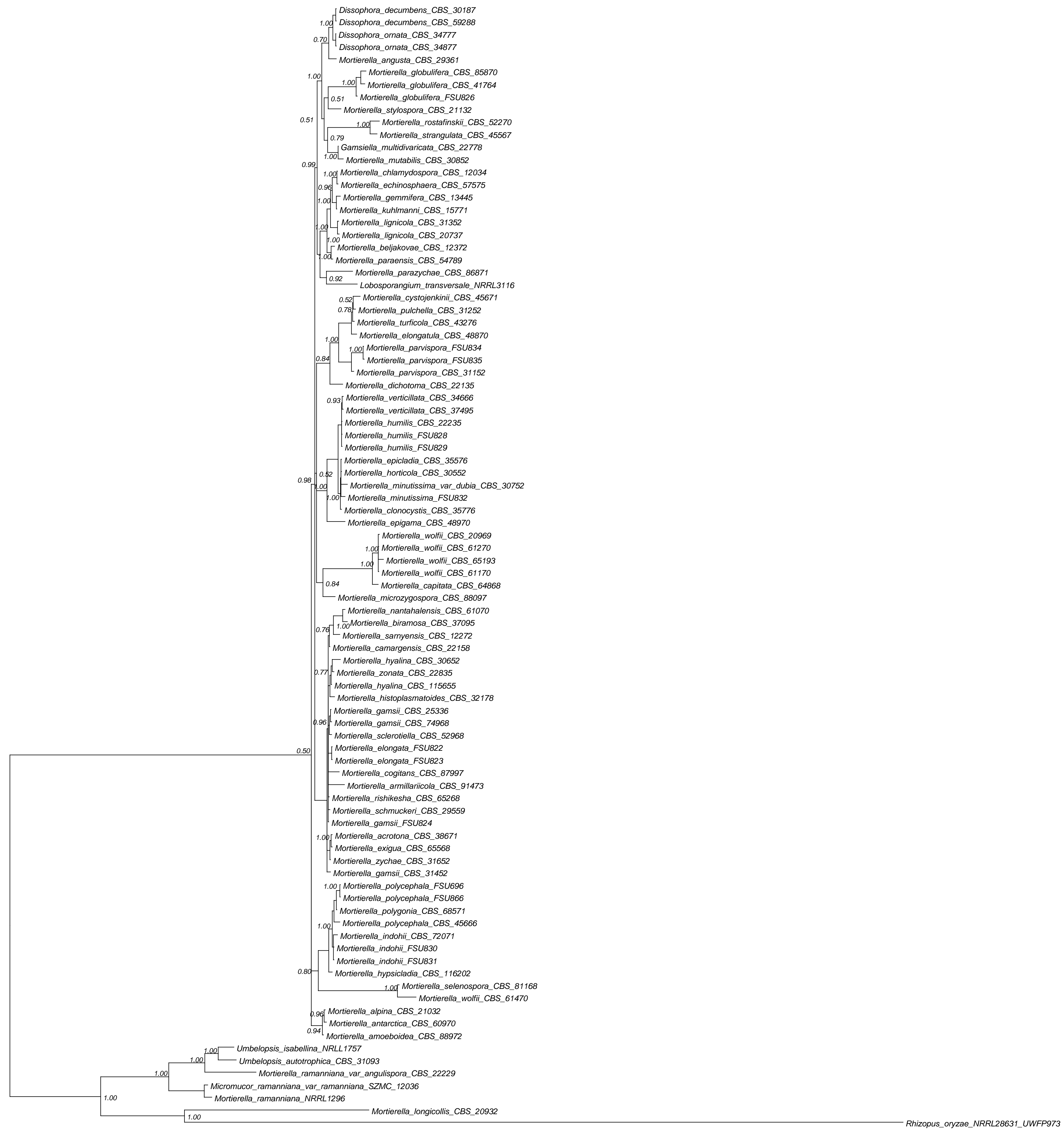

Supplement: Figure S2 — Consensus tree computed from 4000 post-burn-in trees sampled by using the GBlocks curated ITS region, the nuclear ribosomal large (LSU) and small (SSU) subunits without the indel-matrix. (PDF) [file pone.0027507.s002.pdf]

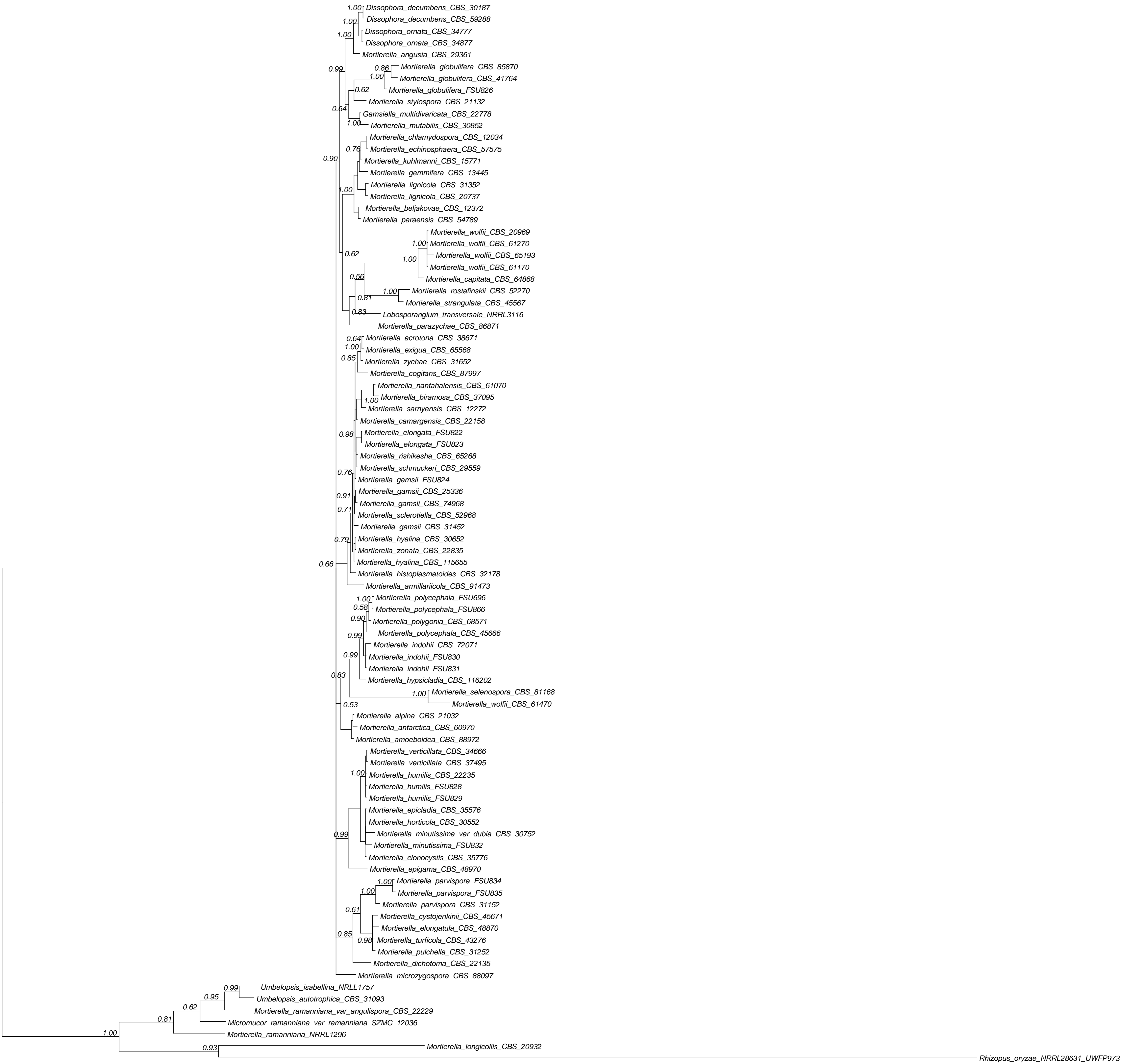

Supplement: Figure S3 — Consensus tree computed from 4000 post-burn-in trees sampled by using the nuclear ribosomal large (LSU) and small (SSU) subunits without the indel-matrix. (PDF) [file pone.0027507.s003.pdf]

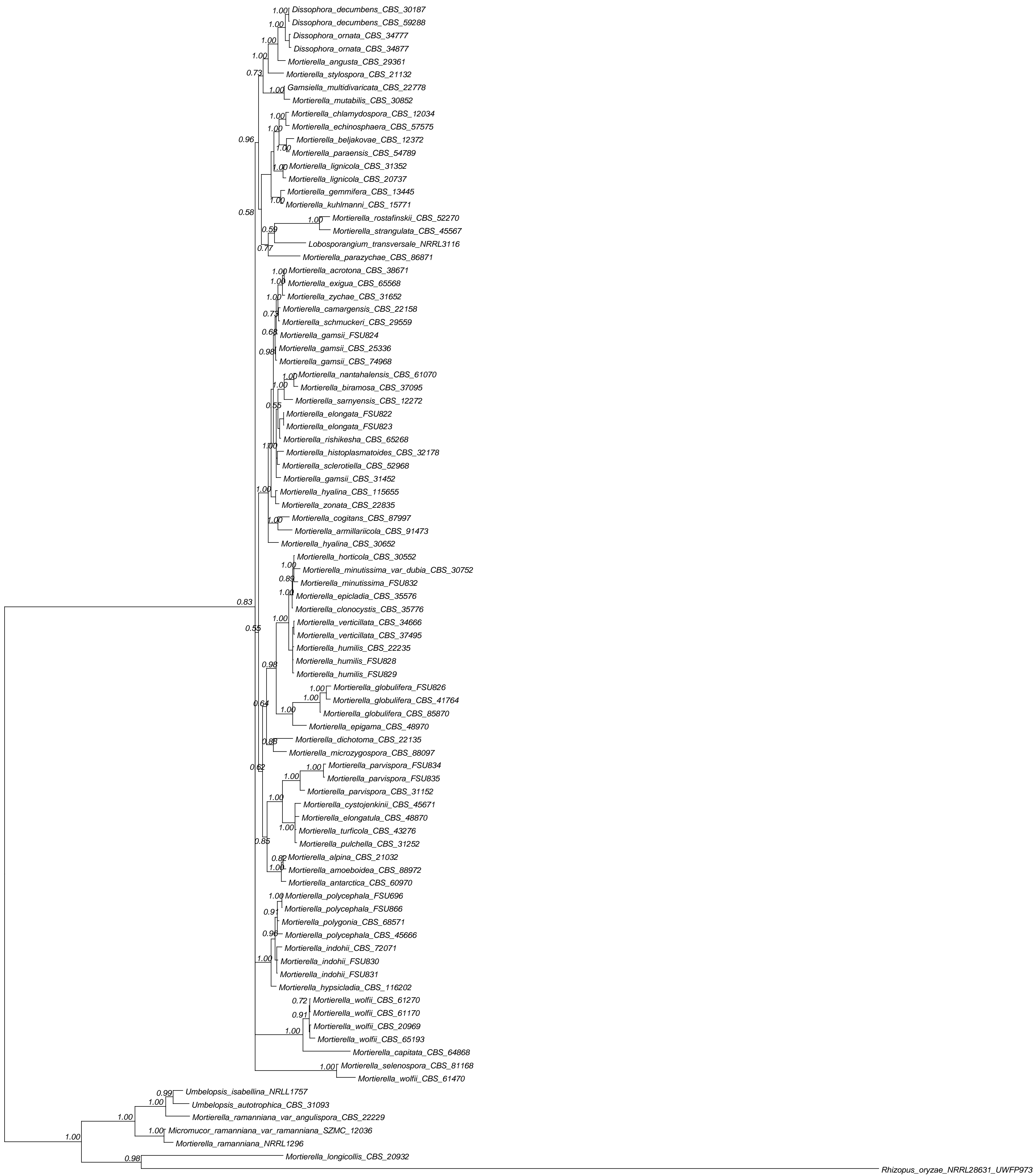

Supplement: Figure S4 — Consensus tree computed from 4000 post-burn-in trees sampled by using the nuclear ribosomal large (LSU) and small (SSU) subunits and the indel-matrix. (PDF) [file pone.0027507.s004.pdf]
