# Supplementary material for: Data Partitions, Bayesian Analysis and Phylogeny of the Zygomycetous Fungal Family Mortierellaceae, Inferred from Nuclear Ribosomal DNA Sequences
Source: PLoS One. 2011 Nov 10;6(11):e27507. doi: 10.1371/journal.pone.0027507 (PMC3213126; doi:10.1371/journal.pone.0027507)
Supplement: Table S1 — Fungal strains included in this study, their collection numbers and type status, and the accession numbers of the sequences deposited in GenBank. (PDF) [file pone.0027507.s005.pdf]

Table S1. Fungal strains included in this study, their collection numbers and type status, and the accession numbers of the sequences deposited in GenBank.

Taxon name	Strain number[a]		GenBank accession no.		
		Status[b]	SSU	ITS	LSU
Dissophora decumbens Thaxt.	CBS 301.87	–	HQ667439	HQ630275	HQ667354
D. decumbens	CBS 592.88	–	HQ667440	HQ630276	HQ667355
D. ornata W. Gams	CBS 347.77	*M. ornata* holotype	HQ667441	HQ630277	HQ667356
D. ornata	CBS 348.77	*M. ornata* holotype	HQ667442	HQ630278	HQ667357
Gamsiella multidivaricata Benny & M. Blackw.	CBS 227.78	*M. multidivaricata* isotype	HQ667475	HQ630314	HQ667391
Lobosporangium transversale M. Blackw. & Benny	NRRL3116	–	HQ667488	–	HQ667404
Micromucor ramannianus var. *ramannianus* (Möller) Arx	SZMC 12036	–	HQ667452	HQ630288	–
Mi. ramannianus	NRRL1296	–	–	HQ630289	HQ667366
Mortierella acrotona W. Gams	CBS 386.71	type	HQ667489	HQ630328	HQ667405
M. alpina Peyronel	CBS 210.32	*M. renispora* authentic	HQ667501	HQ630345	HQ667421
M. amoeboidea W. Gams	CBS 889.72	type	HQ667502	HQ630346	HQ667422
M. angusta Linnem.	CBS 293.61	*M. polycephala* var. *angusta* neotype	HQ667443	HQ630279	HQ667358
M. antarctica Linnem.	CBS 609.70	type	HQ667503	HQ630347	HQ667423
M. armillariicola W. Gams	CBS 914.73	type	HQ667446	HQ630282	HQ667361
M. beljakovae Milko	CBS 123.72	type	–	HQ630352	HQ667428
M. biramosa Tiegh.	CBS 370.95	*M. wuyishanensis* type	HQ667473	HQ630312	HQ667389
M. camargensis W. Gams & R. Moreau	CBS 221.58	type	HQ667492	HQ630331	HQ667408
M. capitata Marchal	CBS 648.68	*M. vesiculosa* type	–	HQ847055	HQ667418
M. chlamydospora (Chesters) Plaäts–Nit.	CBS 120.34	*Azygozygum chlamydosporum* syntype	HQ667508	HQ630354	HQ667430
M. clonocystis W. Gams	CBS 357.76	type	HQ667479	HQ630318	HQ667395
M. cogitans Degawa	CBS 879.97	type	HQ667445	HQ630281	HQ667360
M. cystojenkinii W. Gams & Veenb.–Rijks	CBS 456.71	type	HQ667504	HQ630348	HQ667424
M. dichotoma Linnem.	CBS 221.35	syntype	HQ667477	HQ630316	HQ667393
M. echinosphaera Plaäts–Nit.	CBS 575.75	holotype	–	GU559985	HQ667431
M. elongata Linnem.	FSU 822	–	HQ667494	HQ630336	HQ667412
M. elongata	FSU 823	–	HQ667495	HQ630337	HQ667413
M. elongatula W. Gams & Domsch	CBS 488.70	type	HQ667505	HQ630349	HQ667425
M. epicladia W. Gams & Emden	CBS 355.76	type	HQ667480	HQ630319	HQ667396
M. epigama W. Gams & Domsch	CBS 489.70	type	HQ667453	HQ630290	HQ667367
M. exigua Linnem.	CBS 655.68	type	HQ667490	HQ630329	HQ667406
M. gamsii Milko	CBS 253.36	*M. spinosa* syntype	HQ667497	HQ630339	HQ667415
M. gamsii	FSU 824	–	HQ667498	HQ630341	HQ667417
M. gamsii	CBS 749.68	type	–	HQ630340	HQ667416
M. cf. *gamsii* Milko	CBS 314.52	*M. spinosa* syntype	HQ667468	HQ630307	HQ667384
M. gemmifera M. Ellis	CBS 134.45	type	HQ667456	HQ630293	HQ667371
M. globulifera O. Rostr	CBS 417.64	*M. ericetorum* type	–	HQ847056	HQ667370
M. globulifera	CBS 858.70	neotype	HQ667454	HQ630291	HQ667368
M. globulifera	FSU 826	–	HQ667455	HQ630292	HQ667369
M. histoplasmatoides W. Gams	CBS 321.78	type	HQ667470	HQ630309	HQ667386
M. horticola Linnem.	CBS 305.52	syntype	HQ667483	HQ630322	HQ667399
M. humilis Linnem.	CBS 222.35	syntype	HQ667485	HQ630325	HQ667401
M. humilis	FSU 828	–	HQ667486	HQ630326	HQ667402
M. humilis	CBS 745.68	–	HQ667487	HQ630327	HQ667403
M. hyalina (Harz) W. Gams	CBS 306.52	*M. hygrophila* var. *minuta* syntype	HQ667460	HQ630297	HQ667376
M. cf. *hyalina* (Harz) W. Gams	CBS 115655	*Hydrophora hyalina* isotype	HQ667509	HQ630355	HQ667432
M. hypsicladia Degawa & W. Gams	CBS 116202	type	–	HQ630302	HQ667379
M. indohii C.Y. Chien	FSU 830	–	EU736291	HQ630299	EU736318
M. indohii	FSU 831	–	HQ667462	HQ630300	HQ667438
M. indohii	CBS 720.71	isotype	HQ667461	HQ630298	HQ667377

M. kuhlmanni W. Gams	CBS 157.71	type	HQ667457	HQ630294	HQ667372
M. lignicola (G.W. Martin) W. Gams & R. Moreau	CBS 207.37	*Haplosporangium lignicola* type	HQ667511	HQ630357	HQ667435
M. lignicola	CBS 313.52	*M. sepedonioides* type	HQ667510	–	HQ667434
M. longicollis Dixon–Stew.	CBS 209.32	authentic	HQ667451	HQ630287	HQ667365
M. microzygospora Degawa	CBS 880.97	type	HQ667478	HQ630317	HQ667394
M. minutissima Tiegh.	FSU 832	–	EU736292	HQ630324	EU736319
M. minutissima var. *dubia* Linnem.	CBS 307.52	syntype	HQ667484	HQ630323	HQ667400
M. mutabilis Linnem.	CBS 308.52	syntype	HQ667476	HQ630315	HQ667392
M. nantahalensis C.Y. Chien	CBS 610.70	type	HQ667472	HQ630311	HQ667388
M. paraensis Pfenning & W. Gams	CBS 547.89	type	–	HQ630353	HQ667429
M. parazychae W. Gams	CBS 868.71	type	HQ667447	HQ630283	HQ667362
M. parvispora Linnem.	CBS 311.52	syntype	HQ667458	EU484279	HQ667373
M. parvispora	FSU 834	–	HQ667459	HQ630295	HQ667374
M. parvispora	FSU 835	–	–	HQ630296	HQ667375
M. polycephala Coem.	CBS 456.66	–	–	HQ630335	HQ667411
M. polycephala	FSU 696	–	HQ667493	HQ630332	HQ667409
M. polycephala	FSU 866	–	–	HQ630333	HQ667410
Mortierella polygonia W. Gams & Veenb.–Rijks	CBS 685.71	type	HQ667463	HQ630301	HQ667378
M. pulchella Linnem.	CBS 312.52	authentic	HQ667507	HQ630351	HQ667427
M. ramanniana var. *angulispora* (Naumov) Linnem.	CBS 222.29	neotype	HQ667450	HQ630286	HQ667364
M. rishikesha B.S. Mehrotra & B.R. Mehrotra	CBS 652.68	type	HQ667469	HQ630308	HQ667385
M. rostafinskii Bref.	CBS 522.70	neotype	HQ667512	HQ630358	HQ667436
M. sarnyensis Milko	CBS 122.72	type	HQ667474	HQ630313	HQ667390
M. schmuckeri Linnem.	CBS 295.59	syntype	HQ667496	HQ630338	HQ667414
M. sclerotiella Milko	CBS 529.68	type	HQ667471	HQ630310	HQ667387
M. selenospora W. Gams	CBS 811.68	type	HQ667499	HQ630343	HQ667419
M. strangulata Tiegh	CBS 455.67	neotype	HQ667513	HQ630359	HQ667437
M. stylospora Dixon–Stew.	CBS 211.32	type	HQ667444	HQ630280	HQ667359
M. turficola Y. Ling	CBS 432.76	neotype	HQ667506	HQ630350	HQ667426
M. verticillata Linnem.	CBS 346.66	*M. marburgensis* syntype	HQ667481	HQ630320	HQ667397
M. verticillata	CBS 374.95	*Haplosporangium attenuatissimum* type	HQ667482	HQ630321	HQ667398
M. wolfii B.S. Mehrotra & Baijal	CBS 209.69	–	HQ667464	HQ630303	HQ667380
M. wolfii	CBS 611.70	–	HQ667467	HQ630306	HQ667383
M. wolfii	CBS 612.70	–	HQ667465	HQ630304	HQ667381
M. wolfii	CBS 651.93	–	HQ667466	HQ630305	HQ667382
Mortierella cf. *wolfii*	CBS 614.70	–	HQ667500	HQ630344	HQ667420
M. zonata Linnem.	CBS 228.35	type	–	HQ630356	HQ667433
M. zychae Linnem.	CBS 316.52	type	HQ667491	HQ630330	HQ667407
Rhizopus oryzae Went & Prins. Geerl.	NRRL28631	–	AF113440	–	AY213626
Umbelopsis autotrophica (E.H. Evans) W. Gams	CBS 310.93	*Mortierella ramanniana* var. *autotrophica* type	HQ667449	HQ630285	HQ667363
U. isabellina (Oudem.) W. Gams	NRLL1757	–	HQ667448	HQ630284	–

[a] Abbreviations: CBS, Centraalbureau voor Schimmelcultures, Utrecht, The Netherlands, FSU, Fungal Reference Centre, Jena, Germany, NRRL, Agricultural Research Service Culture Collection, Peoria, Illinois, U.S.A., SZMC, Szeged Microbial Collection, Hungary.

[b] The status of the strains is presented as to be found in Mycobank (http://www.mycobank.org/).
